# Some Results from the Abuse of Quinine

**Published:** 1890-11

**Authors:** J. T. Martin

**Affiliations:** Woodland, Cal.


### SOME RESULTS FROM THE ABUSE OF QUININE.

[Read before the California State Homœopathic Society, May 14th, 1890.]

J. T. Martin, M. D., Woodland, Cal.

We are not going to claim any special originality in the choice of subjects, or in the matter presented. It may seem a little presumptuous, however, to undertake one capable of so great and indefinite expansion; but be that as it may, what we can't handle of it, we most sincerely hope you may use up after we are through. The more we study it the more it grows in proportions, and the harder it is to select the material for a short essay. So it will not be at all strange if there is some left for you to digest. To do the subject full justice would occupy too much of your time.

We merely intend to present to you a few facts so combined that they ought to elicit a more than passing notice from all interested in this investigation. It would doubtless be better for suffering mankind the world over if we all gave more attention to the over use of drugs in general and this one in particular.

Men and even physicians, too, often are not independent thinkers enough to decide questions of therapeutics for themselves; but depend too largely upon the dictum of some supposed authority, without following his course of reasoning in the only way that would prove his conclusions either true or false. It seems to me a self-evident proposition that whenever a medicine is used in a sufficient quantity to suppress a disease from which a patient is suffering, it is used abusively, by causing in many instances a much more serious disease instead of curing the one intended.

The belief expressed in these words, "If a little is good, much is better," has done a world of harm to unsuspecting mankind by inducing them to load up with medicines, charge after charge, expecting that in some way the disease would be fired out by one of these potent charges. Its cognate, "It won't do any harm, if it don't do any good," may, in the light of future years, be equally fertile in producing mischief.

We take from our own experience a case that seems bristling full of suggestions to the thoughtful student. It is this: Some two years ago a little boy was brought to my office with a slight attack of malarial fever, which possessed no alarming symptoms. Indeed, so light was the fever that the child was allowed to run at large during the forenoon, but would lie down in the after part of the day. He went on this way for several days, till one day his mother allowed him to eat some watermelon, which caused his fever to increase quite fast. At this juncture the friends wanted a Quinine tonic, which I could not advise, and they sent to an old-school physician, obtained one and gave it according to directions, with the result that the fever was suppressed in a short time, leaving the child, as they supposed, well of his fever, and with nothing left to do but to recover his usual strength and vigor. The interesting part of the case, however, is the subsequent history. After relief from the fever he did not, as was supposed he would, recover his wonted health; but drooped, and was very languid for a month or so, when he was suddenly seized with an acute attack of cerebro-spinal meningitis, and died in a few days. After observing this case, and noting the result, and believing, as we did, that the suppression of the malarial attack was the direct cause of the meningitis, we hunted the current literature of the day at our command, to find evidence to support or disprove our theory. In the meantime, a friend who belonged to the old school, speaking of the use of Quinine in cerebro-spinal meningitis, said he never saw a case of this disease recover that had been treated with that drug. He had had quite a large experience with this disease in its various forms.

I have in mind a case of cerebro-spinal meningitis treated by scientific medicine that, although much prolonged, was progressing toward recovery, when the attending physician thought to help him regain his lost strength and usual vitality by spurring up the life-forces to do better and quicker work by means of a Quinine tonic.

After using this medicine for not more than a week, he suddenly took a relapse and died. I have often thought what a consolation it certainly must be to the poor, weary doctor that the people in general attribute such results to the over-ruling Providence of an allwise God. The burden is lightened. The friends are satisfied and the doctor very likely lands the next one in the same place.

Dr. George B. Wood, in his work, Vol. I, edition 1860, says, in speaking of experiments upon dogs, even meningitis has in some relatively few instances been brought on by very large doses. Again, on page 249, same volume, the doctor says cases are on record in which death has occurred from inflammation of the brain from the excessive use of Quinine. And on page 250 he goes on to say that another danger from Quinine is the great and secondary prostration from enormous doses, which in persons already feeble may possibly, in some instances, prove fatal. In the transactions of the American Neurological Society, reported in the *Medical Record* of July 27th, 1889, Dr. Prince, of Boston, speaking of the frequency of tabes and other spinal degenerations in cases which also presented a history of malaria, said the first case he had recorded was one of locomotor ataxia, with typical tabetic crisis before each malarial chill. Altogether, the author reported in detail six cases showing the co-existence of tabes with malaria, and six cases where malarial history was associated with sclerosis. Besides these he had notes on three other cases of multiple sclerosis, two of locomotor ataxia, and one of lateral sclerosis with a similar relation.

Dr. Erbb following, stated that tabes may occur as a sequel of intermittent fever, but neglected to add, when suppressed by Quinine.

Dr. Prince again entering the discussion, stated that he had documentary evidence of the pre-existence of malarial poison in all his cases taken from the army register, as they were all old soldiers.

We wish we had the documentary evidence at hand to show that these old soldiers took plenty of Quinine when this malarial poison first made its appearance. But we have not, neither have we the least doubt that such a record exists, and were I able to place it before you to-day you would then see that these cases just mentioned were all suffering from suppressed intermittent. Most of you, however, will agree with me in the following proposition: that if we have an old-school physician and a case of malarial fever, said case is as sure to receive a plentiful supply of Quinine, as that the doctor lives to get there. It is hardly necessary for me to tell you that most all our army physicians belong to the old school, and, therefore, would fulfill the conditions of this proposition. Such discussions as were reported from the American Neurological Society ought to result in ultimate truth and be a lasting benefit to shaking humanity. Let them become more and more general until the glorious sunlight of medical truth shall shine full in their faces. Some two years ago last fall a gentleman who was working on the Seventy-six Ditch Company's ditch in Fresno County, called at my office to be relieved from a condition very much resembling paraplegia. Sensation seemed to be normal, but voluntary motion was very much impaired. His joints were stiff but not swollen. He could not always make the motions with his limbs that was intended. Could not raise his arm to his head or use it in conveying food to his mouth. He also had but little use of his lower extremities. They were so unmanageable that a little unevenness in his path or a roll in the carpet would cause him to tumble down, from which position he could not rise without help. In addition to the above symptoms he had a tremor very much like an alcoholic tremor and very distressing to him. We immediately wanted to know why this man was in such a distressing condition. Why had he not been relieved from what seemed to have been a very simple attack of intermittent fever.

During the course of the investigation we soon learned why. He had been working near water running through a newly-dug irrigation ditch and had contracted chills and fever, the paroxysm occurring between ten and eleven A. M., with thirst during the chill; headache, aching of bones, with nausea and vomiting as soon as the chill was going off and the heat coming on. During the heat, headache and thirst increased, and was very much relieved during the perspiration. He had been treated with Quinine for three months, which resulted in the condition just stated. It is not necessary to follow in detail the treatment of this case, which lasted some six weeks, but suffice it to say that Nat-mur., the remedy first and originally indicated, caused the return of chills. The more he shook the more he limbered up, till he recovered his usual strength and agility, when the chills had entirely left. They have not returned up to the time of this writing, although he has been working all the time in the same place, under the same circumstances, and subject to the same exposures as when first attacked.*

In the early spring of 1889, I was called to the bedside of a young man about seventeen years of age, suffering from general anasarca. Face bloated, hands, feet, extremities as well as his entire body were all swollen to their fullest extent. In fact, he was the worst bloated specimen of humanity that I ever saw. He was exceedingly nervous, possessing a nervous tremor very much like that which results from intoxication. He suffered very much from pains of a rheumatic nature in various parts of the body that were very distressing, and possibly due to extensive infiltration into the cellular tissue.

He passed large quantities of blood with each evacuation of the bladder, mixed with the urine in such a way as to leave no doubt that it came from the kidneys, and, of course, with so much blood there was albumen also. The previous history of this case, which I obtained from the mother, is very interesting and worthy of study.

* This case reported that the chills returned some three months subsequent to the reading of this paper.

The spring after he was three years old, he had an attack of malarial fever with no indications of chills. This was called and treated for typho-malarial fever, and promptly suppressed by Quinine, but returned the next spring, and was completely squelched with the same drug as before. It was bold enough to return on the third spring, when the same guns, similarly loaded, were brought to bear upon it with equally good result, and so it returned every spring for fourteen years, and was as promptly subdued by the same approved methods. Finally, we were called in to subdue the last attack, when his poor, weak body was so full of the material fired there to kill the intruder that it was absolutely impossible to make it hold any more. He went on getting better, then worse, till his lungs filled up and he died with what the doctors called Bright's disease. Speaking of the abuse of China, Hahnemann says: "Note their earthy complexion, their puffy faces, the dullness of their eyes; see how oppressed is their breathing; how hard and extended their epigastrium; how tensely swollen their loins; how miserable their appetite: how perverted their tastes; how painful and oppressed their stomachs are by all food;" and so he goes on to expatiate on the grosser effects of this drug, that, when rightly used, is such a blessing to mankind. The picture that Hahnemann has given us of the over-use of Quinine is remarkably similar to the case just reported that had been treated so long with the sulphate.

Had the stupid doctor ever read Hahnemann's preface to his article on Cinchona, he could have seen that the original disease had long ago ceased to exist and he had to deal with the drug disease cinchonism, which is much harder to cure than the original trouble. He might then have been able to relieve the sufferer, instead of hurrying him on to a premature and untimely grave. We may draw a few pertinent conclusions from the facts we have herein collected. First, Quinine has a specific action on the spinal marrow and nerves, and cerebro-spinal meningitis may be brought on either by an overdose of this drug, or by the suppression of an attack of malarial fever by it. This, we think, is more liable to occur in children and younger people than in those of more mature years. It comes on suddenly, and is not amenable to ordinary old-school treatment; whether it would be to good homœopathic treatment or not I am at present not able to say. Second, the suppression of intermittent fever will produce locomotor ataxia, sclerosis, tabes, paraplegia, and other forms of spinal degenerations. These forms of disease are more liable to occur in adults and persons of middle age and older. Such are more able to withstand the more immediate effects of the drug, and we find the spinal troubles coming on long after the attack has been forgotten. It will kill at long range as well as short, and give your victim a chance to get out of your sight before he lies down to peaceful slumbers or is gathered unto his fathers. Third, it will produce, through its action on the liver, kidneys, and spleen, a condition very closely resembling general anasarca that is very distressing, and may, we think, if followed up, in many cases prove fatal. This latter trouble comes on more insidiously and is fully developed before any one is aware of its true nature, and when organic change has taken place, you will find it most impossible to relieve. The weight of the drug is still there, and ready to render futile your best efforts. It is a good deal like digging in the quicksands, the more you throw out the more will run in.

Possibly you may have noticed the increased number of suicides noted in the secular press of the country during the late epidemic of influenza. On one day, in the early part of January, there were eighty-two bodies of suicides reported in the morgue in New York City alone. They were largely treated with Quinine and antipyrine, and if you will take the trouble to read over the pathogenesis of the former drug, you will be enabled easily to account for this increase in self-destruction. "It makes him gloomy, fills him with thoughts of suicide, he wants to destroy himself and so on."

The time is fast approaching when intelligent people will resist the doctor in his efforts to produce cinchonism to cure ague, or any other disease for that matter. We would not do the subject anything like justice did we not speak of the tonic use of this drug, which we think is very pernicious. For a time it seems to build up the system and make the patient happy in the thought that he is speedily to be restored to health, but this too often proves to be only a delusion and a snare, it lets him down lower than he was before. If the patient be of a good and strong constitution, there may be no immediately bad effects noted—like those old soldiers who fought so valiantly for the preservation of the Union, escaping the horrors of a death on the battlefield only to die years afterward by that slow and torturing death that comes on so gradually, and is the result of a misguided practice that ought to seek only the good of its fellow-man. Taken for the relief of a cold, its first effect is to create a feeling of pleasure and exhilaration, with a sensation at least of partial relief from trouble. Bnt when the next cold comes on it demands the same relief, and the next, and so on. Then they come on oftener and still oftener, till one is continuously taking tonic for colds. Most of you who live in either the San Joaquin or Sacramento valleys have in all probability seen cases of this sort. I have. This taking Quinine for every little ailment produces a condition of the system that howls and cries aloud for more and more of the stuff, and will continue to do so till the demand either is satisfied, squelched with a suitable antidote, or till the stomach rises up to reject the intruder.

In cases where persons are somewhat feeble and have used this drug for some time, and for any reason leave off its use, it often produces a profound prostration that may in some instances prove fatal. This may also be the case when doses very much too large have been taken. The use of the drug purely as a tonic is not without its bad results, and is liable to do considerable harm, because the patient is led to think he is taking something very simple and capable only of giving him health and strength. This often proves a disappointment, and the poor patient seeks another tonic, but gets one with the same base and believes himself taking an entirely new mixture.

Should a patient who has been so treated come to any of you for treatment, he will likely tell you that he has only taken a little tonic—just as if it was the simplest thing in the world. We would like to give you some facts in regard to the use of this drug in typhoid and typho-malarial fevers, but our paper has already grown to such length that we would occupy more than our share of your time.

